# Aortic, musculoskeletal and organ characteristics on computed tomography in knee osteoarthritis – an explorative study in the IMI-APPROACH cohort

**DOI:** 10.1007/s00296-025-05817-3

**Published:** 2025-02-24

**Authors:** Netanja I. Harlianto, Pim A. de Jong, Wouter Foppen, Edwin Bennink, Stijn Bunk, Simon C. Mastbergen, Adriane D. M. Vorselaars, Mareye Voortman, Margreet Kloppenburg, Francisco J. Blanco, Ida K. Haugen, Francis Berenbaum, Karteek Popuri, Mirza Faisal Beg, Mylène P. Jansen

**Affiliations:** 1 Department of Radiology, University Medical Center Utrecht and Utrecht University, Heidelberglaan 100, Utrecht, GA 3508 The Netherlands; 2 Image Sciences Institute, University Medical Center Utrecht and Utrecht University, Utrecht, The Netherlands; 3 Rheumatology and Clinical Immunology, University Medical Center Utrecht and Utrecht University, Utrecht, The Netherlands; 4 Division of Heart and Lungs, University Medical Center Utrecht and Utrecht University, Utrecht, The Netherlands; 5 ILD Center of Excellence, Department of Pulmonology, St. Antonius Hospital, Nieuwegein, The Netherlands; 6 Department of Rheumatology, Leiden University Medical Center, Leiden, The Netherlands; 7 Clinical Epidemiology, Leiden University Medical Center, Leiden, The Netherlands; 8 Departamento de Fisioterapia y Medicina, Grupo de Investigación de Reumatología (GIR), Centro de Investigación CICA, Universidad de A Coruña, A Coruña, Spain; 9 INIBIC – Complejo Hospitalario, Universidad de A Coruña, A Coruña, Spain; 10 Center for Treatment of Rheumatic and Musculoskeletal Diseases (REMEDY), Diakonhjemmet Hospital, Oslo, Norway; 11 Department of Rheumatology, AP-HP Saint-Antoine Hospital, INSERM, Sorbonne University, Paris, France; 12 Department of Computer Science, Memorial University of Newfoundland, St. John’s, Canada; 13 School of Engineering Science, Simon Fraser University, Vancouver, Canada

**Keywords:** OA, Body composition, Adipocyte, Atherosclerosis, Pulmonary, Breathing

## Abstract

The systemic associations with knee osteoarthritis (KOA) are incompletely understood. This study explores aortic disease, musculoskeletal and organ findings in patients with KOA in relation to their symptoms or radiographic abnormalities. Full body computed tomography (CT) scans of 255 IMI-APPROACH participants were investigated using an automated analysis of multislice CT (Voronoi Health Analytics) that extracts aortic size and calcifications, and volumes and densities of bones, muscles, fat compartments and thoracic and abdominal organs. The CT measurements were primarily related to KOA as measured with Western Ontario and McMaster Universities Osteoarthritis Index (WOMAC), visual scores and automated knee radiograph analysis of osteophytes, bone sclerosis and joint space width. The median age was 67 years, body mass index (BMI) 26.8 kg/m^2^ and 78% were female. About half had Kellgren-Lawrence grade ≥ 2. Larger knee osteophyte area was associated with a larger aortic volume (R_Spearman_=0.21,*P* = 0.001), which can be due to elongation or dilatation. We observed an association between more symptoms and increased psoas (R_Spearman_=-0.23,*P* < 0.001) and lower leg (R_Spearman_=-0.23,*P* < 0.001) muscle density, suggesting less microscopic muscle fat. Symptomatic KOA was associated with substantially lower lung volume (771 ml difference between 50% worst and 50% best WOMAC), but not with visible lung disease. Lung volume and density were significantly associated with the physical functioning WOMAC component. These associations remained significant after adjustment for age, sex and BMI. KOA is associated with significant systemic changes, including altered aortic and organ volumes. These correlations suggest that KOA’s impact may extend beyond the joints. Future research should explore the causal relationships and therapeutic implications associations.

**Supplementary Information:**

The online version contains supplementary material available at 10.1007/s00296-025-05817-3.

## Introduction

Knee osteoarthritis (KOA) is a common disease with an increasing prevalence in the aging population. It leads to a substantial loss of quality of life. Evidently, KOA is multifactorial, but the etiology is incompletely understood and currently there is no medication that slows the progression of joint degeneration. It is recognized that KOA is more than cartilage loss, as the whole knee joint with all its tissues is involved in the disease process. Even beyond the knee there is a systemic contribution to the disease, which may differ between males and females [1]. High body mass index (BMI) and inflammatory pathways are associated with KOA [2]. However, BMI is a difficult to interpret and data suggest that more detailed analysis of body composition can add our understanding of the etiology of KOA, and investigators and clinicians have been warned for too much focus on body weight [3–5]. Also, the role of atherosclerosis and arterial calcification in KOA remains uncertain [6]. Outside the vascular and musculoskeletal systems much less is known on the abdominal and thoracic organs in KOA patients.

One of the aims of the Innovative Medicine Initiative Applied Public-Private Research enabling OA Clinical Headway (IMI-APPROACH) study was to identify (bio)markers for cartilage loss and its progression rate in the knee. For that purpose, it was needed to have a surrogate measure for cartilage loss in other joints, for which whole body was CT chosen [7]. In the present study we used these whole body CT scans to comprehensively explore, aortic disease, musculoskeletal characteristics and organ sizes in patient with KOA according to clinical criteria. Exploring organ characteristics from full body CT scans should enable a holistic understanding of KOA, potentially revealing novel biomarkers and systemic involvement that go beyond the localized joint pathology traditionally associated with the disease. Symptomatic and radiographic KOA severity were associated to whole body CT characteristics as a hypothesis generating investigation.

## Methods

### Patient population

The IMI-APPROACH study has been described in detail in a previous publication [8]. In short, patients in the IMI-APPROACH cohort were included with predominantly tibiofemoral KOA and satisfying the clinical American College of Rheumatology (ACR) classification criteria for KOA; being able to walk unassisted; ≥18 years of age; high probability of progression of KOA. A total of 297 participants were included and followed for two years in five hospitals in France (Saint-Antoine hospital, Paris), The Netherlands (UMC Utrecht and Leiden UMC), Norway (Diakonhjemmet hospital, Oslo), and Spain (SERGAS, A Coruna). The study was conducted in accordance with the Declaration of Helsinki (as revised in 2013). The study was approved by the regional ethics committees and Institutional Review Boards and informed consent was taken from all individual participants.

### Baseline data collection

Baseline data collection included BMI (calculated using each participant’s height and weight), blood pressure, and smoking status assessment defined as never versus ever smoker. In addition, the Western Ontario and McMaster Universities Osteoarthritis Index (WOMAC) and its three components (pain, stiffness, function) were calculated, all expressed from 0 to 100 with 100 indicating no complaints, using the Knee Injury and Osteoarthritis Outcome Score questionnaire. Finally, according to the Buckland-Wright protocol [9], weight-bearing anteroposterior knee radiographs were obtained, on which Kellgren-Lawrence grading (KLG) was performed by one experienced observer as a measure of (structural) KOA severity [8]. For the current study, radiographs of the most affected (index) knee were used.

### CT scanning protocol

Whole-body low-dose CT scans were acquired using six different scanners (GE Lightspeed VCT; Toshiba Aquilion One; Philips Brilliance 64, during the study replaced by Toshiba Aquilion Prime; Siemens Somatom Definiton Edge; Philips IQon Spectral CT 64). The protocol for all centers included a tube voltage of 120 kV and exposure reference of 15 mAs, with a maximum dose of 3.0 mSv for a 70 kg adult. A European Spine Phantom (ESP, QRM Möhrendorf, Germany) was used for cross calibration, ensuring equivalence of CT protocols across scanners. The participants were instructed by the radiographers to hold their breath in inspiration during the scanning procedure. Whole-body iterative reconstructions were performed with medium kernel to obtain 1 mm axial slices.

### CT measurements

We used the medical image Data Analysis Facilitation Suite version 1.3.1 (DAFS, Voronoi Health Analytics, Vancouver, Canada) for automatic body composition measurements. Nonlinear machine learning techniques are employed to process the images, yielding two outcomes: (1) multislice segmentation of various organs and tissues, and (2) annotation of vertebral bones in every picture slice. Dice similarity coefficients between automatic methods and manual segmentation were > 0.95. Details on the segmentation method have been published previously [10–12]. The method quantifies volumes in milliliters and density in Hounsfield Units for the aorta, fat, muscle, bones and various organs. For the vessels, the method segments the entire aorta (ascending aorta till aortic bifurcation) and provides its volume and the Agatston score for the aortic wall calcifications. Volume measurements are based on the number of voxels segmented as a specific tissue, multiplied by the volume of a single voxel. For the musculoskeletal system the method quantifies the volume of visceral adipose tissue, epicardial adipose tissue, paracardial adipose tissue, mediastinal adipose tissue and subcutaneous adipose tissue. Additionally, the left and right psoas, as well as the other skeletal muscles are segmented and summed as total skeletal muscle. For the muscles the volume of muscle tissue, the density of muscle tissue as well as the amount of macroscopic fat is provided. Finally, the skeleton is segmented and volume and density are provided for the whole skeleton and density for the right and left femoral neck.

### KIDA analysis

For quantitative analysis of the knee radiographs, Knee Images Digital Analysis (KIDA) software was used, for which the details have been described previously [13, 14]. One experienced observer measured the whole-joint osteophyte area (mm^2^), subchondral bone density (mm Aluminum equivalent, in reference to an aluminum step wedge), and the minimum joint space width (JSW; mm).

### CT visual scoring of the chest

The CT scans were scored visually by a thoracic radiologist with > 10 years’ experience for various pathologies defined according to Fleischner Society recommendations [15]. The observer was blinded to KOA severity. Arbitrary grading into absent, mild, moderate and severe was used for several lung characteristics. Extrapulmonary scoring consisted of unilateral elevation of the hemidiaphragm and presence of pleural fluid. Intrapulmonary scoring consisted of parenchymal lung disease (emphysema, ground glass, interstitial lung abnormalities, platelike atelectasis or parenchymal bands) or airway diseases (mosaic pattern, wall thickening, bronchiectasis, mucus plugging and inflammation of small airways as tree-in-bud).

### Data analysis

Data are presented as median and interquartile range or number and percentage. Spearman correlation analyses between total body CT measurements and OA measurements total WOMAC and osteophyte sum for the index knee were performed. Further in-depth analysis of significant Spearman correlations was done using multiple linear regression techniques adjusting for age and sex and BMI or subcutaneous fat volume, and hospital site. For the aorta additional adjustments were done for smoking and blood pressure. A chi-square test was used for the association between visual CT scores and WOMAC upper and lower half. The data analysis was done in IBM SPSS version 29.0.0. For the Spearman correlation analysis, a p-value < 0.00167 (Bonferroni corrected p-value for 30 comparisons) was considered significant taking multiple testing into account. For the regression analysis and chi-square tests a p-value < 0.05 was considered significant.

## Results

### Participants

Out of 297 participants in the IMI-approach cohort, 42 participants were excluded due to storage issues, incomplete scans and/or excessive noise. The body composition on CT scans was thus analyzed for 255 participants. Briefly, the median age of the 255 participants was 67 years and the majority were female (78%). About half were current or former smokers and median BMI was 26.8 kg/m^2^. While all had KOA according to the ACR classification criteria, about half had radiographic KOA, defined as KLG ≥ 2. Further details are provided in Table 1.

**Table 1:** Baseline cohort description of 255 knee osteoarthritis patients

Participant characteristics	Value
• Age (years)	67 (62–72)
• Sex female	198 (78.0%)
• Smoking status current or former	132 (53%)
• Height (cm)	166 (159–174)
• Body mass index (kg/m2)	26.8 (24.2–31.8)
• Systolic blood pressure (mmHg)	121 (135-146.5)
**Osteoarthritis severity**	
• Kellgren and Lawrence grade ≥ 2	135 (54%)
• WOMAC	68.8 (53.6–82.3)
• WOMAC pain	70 (55–85)
• WOMAC stiffness	62.5 (50.0–75.0)
• WOMAC function	68.8 (53.8–84.6)
• KIDA sum osteophyte	14.2 (8.4–29.8)
• KIDA joint space width	2.72 (1.71–3.42)
• KIDA bone density	30.2 (27.2–34.4)
**Computed tomography measurements aorta**	
• Aortic volume (ml)	229.8 (192.0-273.1)
• Aortic calcifications (Ln Agatston + 1)	0.27 (0.03–1.14)
**Computed tomography measurements musculoskeletal**	
• Bone volume (ml)	3032 (2740–3519)
• Bone density (HU)	432 (388–478)
• Femoral neck density (HU)	360 (314–409)
• Intramuscular adipose tissue (ml)	1330 (1058–1739)
• Epicardial fat volume (ml)	45.0 (30.7–64.1)
• Pericardial adipose tissue (ml)	94.6 (63.0-145.0)
• Mediastinal adipose tissue (ml)	18.2 (11.7–29.5)
• Subcutaneous adipose tissue (ml)	20,839 (16089–27428)
• Visceral adipose tissue (ml)	2772 (1824–4376)
• Skeletal muscle volume (ml)	5703 (5091–7111)
• Skeletal muscle density (HU)	37.0 (32-5-42.0)
• Psoas volume (ml)	600 (524–719)
• Psoas density (HU)	49.3 (46.0-53.4)
• Lower leg muscle volume (ml)	2867 (2531–3333)
• Lower leg muscle density (HU)	44.8 (40.7–47.7)
• Upper leg muscle volume (ml)	8611 (7450–10341)
• Upper leg muscle density (HU)	36.8 (32.2–40.6)
**Computed tomography measurements organs**	
• Liver volume (ml)	1370 (1187–1607)
• Liver density (HU)	50.3 (36.8–57.5)
• Gall bladder volume (ml)	4.5 (0.6–12.1)
• Heart volume (ml)	629 (551–756)
• Spleen volume (ml)	130.7 (88.1-189.8)
• Kidney volume (ml)	258 (212–320)
• Lung volume (ml)	3951 (2911–4847)
• Lung density (HU)	-761 (-796 - -681)

Data given are median (interquartile range) or number (percentage). Western Ontario and McMaster Universities Arthritis Index (WOMAC). KIDA data and Kellgren and Lawrence grade are provided for the index knee

### Aortic characteristics

In the exploratory study, radiographic knee osteoarthritis (KOA), as assessed by osteophyte area and Kellgren-Lawrence grade (KLG), was significantly associated with increased aortic volume (R_Spearman_ 0.21, *p* = 0.001; Table 2). No evidence was found that this was related to surrogate markers of atherosclerosis such as aortic calcifications as the association between KOA and aortic calcification was not significant. The significant association between aortic volume remained significant after adjustment for age, sex and BMI and also after further adjustment for smoking and blood pressure (Table 3, Supplementary Table S1).

**Table 2:** Spearman correlation between knee osteoarthritis and body composition

	Total WOMAC score		Sum osteophytes	
Determinant	R-Spearman	P-value	R-Spearman	P-value
Age (years)	0.10	0.10	0.058	0.36
Height (cm)	0.17	0.006	0.10	0.11
Body mass index (kg/m2)	-0.35	**< 0.001**	0.11	0.10
**Computed tomography measurements aorta**				
Aortic volume	0.12	0.07	0.21	**0.001**
Ln aortic calcifications	0.13	0.04	-0.017	0.78
**Computed tomography measurements musculoskeletal**				
Bone volume	0.073	0.25	0.017	0.008
Bone density	0.054	0.39	0.074	0.24
Epicardial fat volume	0.017	0.79	-0.003	0.96
Intramuscular adipose tissue	-0.15	0.02	0.084	0.19
Pericardial adipose tissue	-0.12	0.07	0.025	0.69
Subcutaneous adipose tissue	-0.27	**< 0.001**	0.006	0.92
Skeletal muscle volume	0.028	0.66	0.14	0.03
Skeletal muscle density	-0.19	0.002	0.00	0.99
Mediastinal adipose tissue	-0059	0.35	-0.033	0.61
Visceral adipose tissue	-0.18	0.005	0.01	0.88
Femoral neck density	0.12	0.06	0.05	0.42
Psoas volume	0.19	0.003	0.11	0.08
Psoas density	-0.23	**< 0.001**	0.017	0.79
Lower leg muscle volume	0.091	0.16	0.16	0.02
Lower leg muscle density	-0.21	**0.001**	-0.092	0.16
Upper leg muscle volume	0.13	0.04	0.14	0.03
Upper leg muscle density	-0.017	0.78	-0.039	0.54
**Computed tomography measurements organs**				
Gall bladder volume	0.092	0.14	0.032	0.62
Heart volume	-0.001	0.99	0.15	0.02
Liver volume	-0.063	0.32	0.16	0.01
Liver density	0.15	0.02	0.017	0.79
Spleen volume	0.061	0.34	0.13	0.04
Kidney volume	-0.11	0.09	0.10	0.11
Lung volume	0.27	**< 0.001**	0.05	0.44
Lung density	-0.28	**< 0.001**	0.021	0.74

The p-value for 30 comparisons is adjusted to a p-value less than 0.00167. Western Ontario and McMaster Universities Arthritis Index (WOMAC). A lower WOMAC score represents more disability. Ln is natural logarithm. Sum osteophytes are KIDA data provided for the index knee

**Table 3:** Multivariate association between knee osteoarthritis and body composition

	Total WOMAC score	
Determinant	Beta (95%CI)	P-value
Subcutaneous adipose tissue	0.000 (0.000-0.001)	0.130
Psoas density	-0.347 (-0.92- -0.074)	**0.013**
Lower leg muscle density	-0.46 (-0.80- -0.11)	**0.009**
Lung volume	0.002 (0.000-0.004)	**0.021**
Lung density	-0.045 (-0.075- -0.015)	**0.003**
	**Sum osteophytes**	
Aortic volume	0.078 (0.031–0.13)	**0.001**

Western Ontario and McMaster Universities Arthritis Index (WOMAC). Data are adjusted for age, sex body mass index (BMI), and hospital site. Sum osteophytes are KIDA data provided for the index knee. Additional adjustments are provided in the online supplement

### Musculoskeletal characteristics

A significant correlation between clinical KOA severity with BMI and subcutaneous fat volume was observed. A higher BMI and fat volume corresponded with lower WOMAC scores (more complaints) (Table 2). This correlation was not observed for visceral fat. The relation between subcutaneous fat and WOMAC became non-significant when adjusted for BMI in linear regression analysis (Table 3). Like visceral fat, fat around the heart and macroscopic intramuscular fat was not correlated with WOMAC scores. However, an inverse association was observed with psoas and lower leg muscle density where higher density was associated with lower WOMAC scores (more complaints) (Table 2). This remained significant after adjustment for age, sex and BMI. Muscle density is thought to reflect microscopic fat (lower density reflects more microscopic fat), so more complaints were associated with less microscopic intramuscular fat.

For bones and muscles volume no significant correlation with WOMAC or total osteophyte area were observed and neither any significant correlation for bone density could be identified. Similar observations were made for other measurements of radiographic KOA such as KLG, JSW and bone density around the knee (Supplementary Table S2 and S3).

### Organ characteristics

For most organ sizes and density no associations with KOA severity were found, except for a significant association with lower lung volumes and higher lung density, which remained significant after adjusting for age, sex and BMI (Tables 2 and 3, Supplementary Table S2) The median (IQR) lung volume in the 50% with the worst WOMAC scores was 3508 (2530–4674) ml, which compared to 4279 (3547–5103) ml in the 50% with the best WOMAC scores. Thus, the median difference was 771 ml. The associations appeared to be explained by the physical functioning component of WOMAC, more than pain and stiffness (Supplementary Table S4). As lower lung volume can be due to diffuse parenchymal lung disease we performed additional visual lung assessment. However, our visual analyses did not identified more lung disease in participants with more symptomatic KOA (Table 4). Similar for the unilateral hemidiaphragm elevation or pleural fluid, no changes were found. Other possible explanations are difficulty with inhalation due to subcutaneous fat or bilateral diaphragm elevation due to visceral fat. However, the association remained significant after additional adjustments for measures such as BMI, hospital site and subcutaneous and visceral fat volumes (Table 3).

**Table 4:** Clinical knee osteoarthritis severity and pulmonary, pleural and diaphragmatic abnormalities on computed tomography

CT finding	WOMAC worst 50%	WOMAC best 50%	Chi-square p-value
Diaphragm elevation	9 (7%)	11 (9%)	0.63
Pleural fluid	1 (1%)	0 0%)	0.32
Dependent ground glass opacities			0.58
• Mild	29 (23%)	35 (28%)	
• Moderate	49 (39%)	43 (34%)	
• Severe	11 (9%)	7 (6%)	
Interstitial lung disease	7 (6%)	3 (2%)	0.20
Centrilobular emphysema			0.78
• Mild	10 (8%)	12 (10%)	
• Moderate	3 (2%)	5 (4%)	
• Severe	2 (2%)	1 (1%)	
Airway wall thickening			0.49
• Mild	42 (33%)	44 (35%)	
• Moderate	36 (29%)	27 (22%)	
• Severe	6 (5%)	4 (3%)	
Bronchiectasis			0.12
• Mild	11 (9%)	20 (16%)	
• Moderate/severe	1 (1%)	3 (2%)	
Mosaic pattern			**0.03**
• Mild	13 (10%)	17 (14%)	
• Moderate	8 (6%)	21 (17%)	
• Severe	4 (3%)	6 (5%)	
Mucus plugging	7 (6%)	15 (12%)	0.07
Tree in bud pattern	2 (2%)	4 (3%)	0.40
Platelike atelectasis and parenchymal bands	35 (28%)	37 (30%)	0.75

## Discussion

Because the systemic associations of KOA are incompletely understood, aortic disease and musculoskeletal and organ characteristics in relation to radiographic and symptomatic KOA severity was explored. We observed that larger osteophyte area was associated with a larger aortic volume. For symptomatic KOA severity we observed an association with higher muscle density, suggesting less microscopic muscle fat, and an association with lower lung volumes. This requires further investigation but could not be explained by visual lung disease or the amount of adipose tissue. Our findings are explorative and warrant replication, but detailed measurement of fat and muscle compartments beyond BMI in KOA patients is supported by our findings.

We investigated the aortic size and calcification score in our study. The association between KOA and arterial disease remains poorly understood for multiple reasons. One is the difficulty measuring KOA, for example, in our study all participants had ACR defined KOA, while only half had a KLG ≥ 2. The same is true for arterial disease as the measurements are not easy to understand. For example, atherosclerosis can be fatty, fibrofatty or fibrocalcific in nature and arterial stiffening can be due to fibrocalcific atherosclerosis, medial calcification or wall fibrosis. Subsequently there can be differences between females and males [16] and systemic and local effects [6]. Radiographic KOA as measured by osteophyte area was in our explorative study associated with a larger aorta. A larger aorta can be due to elongation (lengthening) and/or dilatation, related to atherosclerosis or low-grade inflammation [17]. However, in the present report we did not find an association with another surrogate of atherosclerosis of the aorta – aortic calcifications. Also, the association remained after additional adjustment of age, sex, smoking status and systolic blood pressure, known risk factors for atherosclerosis. Non-atherosclerotic reasons for increased aortic volume can be connective tissue diseases. Previous work has demonstrated excessive accumulation of fatty acids in the cartilage and synovial membrane of OA joints. This accumulation of fatty acids is mainly due to an alteration in the lipid metabolism of the OA chondrocytes and synoviocytes [18]. This pathogenic process of accumulation of fatty acids in OA cartilage and synovial membrane shows many similarities to the atherosclerosis of vessels [19]. Connective tissue syndromes (Marfan, Ehlers Danlos) are indeed associated with joint laxity and premature OA [20, 21]. In those diseases there is cystic medial degeneration of the aortic wall with loss of smooth muscles and elastic fibers. Another example is aneurysm-osteoarthritis syndrome, an inherited autosomal dominant connective tissue disease caused by SMAD3 mutations [22]. Possibly more common and less severe variations in connective tissue development and regeneration in the populations may explain our findings. Osteophytes maybe an adaptive mechanism to maintain joint stability in the presence of some laxity. Moreover, osteophytes may also be due to inflammatory processes [23]. Finally, chronic mechanical stress on the aortic wall can lead to a loss of elastic fibers and subsequent dilatation. For elastin loss, smoking would be a logical causative factor as smoking enhances elastin degradation, but we did not find evidence for this in our data. Replication and further investigation is required and great care is needed with causal reasoning at the stage.

We explored associations between knee OA and the musculoskeletal system, which has a history in KOA research. There have been ample publications on body composition in knee OA [24–26]. Previous investigators stressed the limitations of focusing solely on weight or BMI and have emphasized differences between males and females in body composition [3–5]. Our data confirm the complexity and differences between BMI and multiple fat compartments. We would have expected an association between visceral fat and KOA, as increased visceral fat promotes chronic inflammation [22], but we did not observe such an association. Of course, our data cannot be considered conclusive in the absence of such an association. Previous work has found that visceral fat is associated with functional pain, but not associated with structural knee OA development [27]. Moreover, a recent pilot study found that serum and synovial adipokines such as chemerin and resistin of visceral fat may play a role in inflammatory KOA changes [28].

We did observe that more severe knee OA symptoms as measured with WOMAC were associated with higher muscle density of the psoas muscle and lower leg skeletal muscles. A higher muscle density indicates less microscopic fat in the muscle in patients with more severe OA symptoms, which was independent of age, sex and BMI. In contrast, a small previous study observed more microscopic fat, at least in the lower leg [29]. Yamouchi et al. [30] studied individual thigh muscles in 50 patients with early and established KOA on MRI. They observed that established KOA was related with more intermuscular adipose tissue. Yet, adipose tissue was not related to KOA grade when analyzing intra-patient left and right differences [30]. The observation could not be explained by muscle atrophy or hypertrophy or by macroscopic intramuscular fat. If technical factors, such as image noise, would be an explanation, a similar association would be expected for other density measures outside the muscle. There may be poorly understood effects of pain on microscopic muscle fat quantity and further investigation using more detailed measurements of (neuropathic) pain and whether it is localized or systemic are of interest for future studies. It could also be that patients with a painful knee use some muscles more often/intense as a coping mechanism or due to physiotherapy and further in dept analysis of mobility, balance and gait are of interest, although in previous studies we mainly observed an association with radiographic KOA and joint structure [31]. In conclusion, it is clear that composition of fat, muscle and bone is complex, and our findings encourage detailed measurement of fat compartments and muscle volume and density beyond BMI, at least in the research setting.

Finally, we explored alteration in organ size and density in relation to knee OA. Clinical knee OA severity as measured with WOMAC was in our exploration significantly related to lower lung volume and higher lung density. This could not be visually explained by intrinsic lung disease, pleural fluid or unilateral diaphragm elevation. Our observation of less mosaic attenuation and mucus may be due to chance and cannot explain the difference in lung volumes. It may be that there is OA associated lung disease below CT resolution or invisible to the eye. Lung fibrosis would be the most likely candidate, but we think this is unlikely as known signs of lung fibrosis were rare in this cohort. It could also be that clinical knee OA is related to difficulty breathing due to adipose tissue. However, the association remained significant after adjustment of subcutaneous fat or BMI. Finally, it may be that breathing itself is different in clinical knee OA patients. A previous population-based study found that subjects with knee OA had lower measured lung function compared to controls, which was not related to the presence of chronic obstructive pulmonary disease [32]. In the same cohort, radiographic OA was associated with the presence of asthma after correcting for age, sex, BMI, and smoking status [33]. Further research could elaborate on measuring breathing muscles such as the intercostal, pectoral and sternocleidomastoid muscles volume, quality and strength by comparing maximum inspiratory and expiratory pressures (MIP/MEP).Moreover, it could be that clinical knee OA patients breathe more superficially, which could be investigated by capnography and chest and abdomen plethysmography. Such measurements could also be done during sleep to exclude conscious control of the breathing during the tests. In our study the association seems to be explained by the physical functioning component of the WOMAC, which is less specific for pure KOA severity. The physical functioning component includes questions such as difficulty using stairs, shopping, heavy domestic duties and light domestic duties. Replication and subsequently more in dept analysis of our exploratory findings is needed.

The weaknesses of our study must be considered. The explorative hypothesis-free investigation can be seen as weakness, and we agree that replication is needed. Also, we did many statistical tests, though we did correct for multiple testing. Second, a limitation is that our cohort primarily consisted of females (78%), with increased BMI (26.8 kg/m2), therefore our data may not be generalizable to persons with other known risk factors for knee OA. Further studies could test whether our findings generalize to other cohorts with different inclusion criteria. Another limitation is the cross-sectional nature of our study, for which residual confounding may still be present, which limits drawing causal conclusions from our findings.

In conclusion, KOA does have systemic associations and our data support an association with fat distribution, aortic volume increases due to dilation and/or elongation and decreased lung volumes. These correlations suggest that KOA’s impact may extend beyond the joints, indicating the potential for systemic biomarkers. Future research should explore the causal relationships and therapeutic implications of these systemic associations to enhance KOA management strategies.

## Electronic supplementary material

Below is the link to the electronic supplementary material.

Supplementary Material 1

## Data Availability

Data are available upon reasonable request from the corresponding author.

## Declarations

### Disclaimer

This manuscript has not been previously archived. No part of this manuscript, including text and graphics, is copied or published elsewhere in whole or in part. Generative AI and AI-assisted technologies were not used in the preparation of this work.

### Note on congress abstracts and publication

This work was not previously presented and/or submitted as conference abstract.
