# Supplementary material for: Aortic, musculoskeletal and organ characteristics on computed tomography in knee osteoarthritis – an explorative study in the IMI-APPROACH cohort
Source: Rheumatol Int. 2025 Feb 24;45(3):62. doi: 10.1007/s00296-025-05817-3 (PMC11850454; doi:10.1007/s00296-025-05817-3)
Supplement: Supplementary file 1 — Supplementary Material 1 [file 296_2025_5817_MOESM1_ESM.docx]

**Supplementary Material**

**Supplementary Table S1**– Osteophyte area sum (KIDA) in relation to aortic volume

| **Independent variable** | **beta** | **P-value** |
| --- | --- | --- |
| Age in years | -0.18 | 0.34 |
| Sex female | -0.94 | 0.79 |
| Smoker current or former | -7.1 | 0.006 |
| Systolic blood pressure | 0.024 | 0.72 |
| Aortic volume | 0.083 | <0.001 |

Data are from one multiple linear regression model

**Supplementary Table S2**- Spearman correlation between additional radiographic knee osteoarthritis markers and body composition

|  | **Kellgren and Lawrence grade** | | **Minimum joint space width (KIDA)** | | **Bone density (KIDA)** | |
| --- | --- | --- | --- | --- | --- | --- |
| **Determinant** | **R-Spearman** | **P-value** | **R-Spearman** | **P-value** | **R-Spearman** | **P-value** |
| Age (years) | 0.10 | 0.13 | -0.13 | 0.04 | -0.14 | 0.002 |
| Height (cm) | -0.001 | 0.99 | 0.11 | 0.09 | 0.069 | 0.28 |
| Body mass index (kg/m2) | 0.13 | 0.04 | -0.009 | 0.88 | 0.22 | **<0.001** |
| **Aortic measurements** |  |  |  |  |  |  |
| Aortic volume | 0.16 | 0.01 | -0.08 | 0.21 | 0.012 | 0.85 |
| Ln aortic calcifications | -0.007 | 0.91 | -0.033 | 0.61 | -0.15 | 0.02 |
| **Musculoskeletal measurements** |  |  |  |  |  |  |
| Bone volume | 0.076 | 0.23 | 0.086 | 0.18 | 0.072 | 0.26 |
| Bone density | 0.012 | 0.85 | -0.007 | 0.91 | 0.16 | 0.01 |
| Epicardial fat volume | -0.009 | 0.89 | 0.058 | 0.36 | -0.12 | 0.05 |
| Intramuscular adipose tissue | 0.11 | 0.08 | 0.025 | 0.70 | 0.19 | 0.003 |
| Pericardial adipose tissue | 0.011 | 0.86 | 0.075 | 0.24 | 0.04 | 0.53 |
| Subcutaneous adipose tissue | 0.062 | 0.33 | 0.038 | 0.55 | 0.074 | 0.24 |
| Skeletal muscle volume | 0.056 | 0.39 | 0.096 | 0.13 | 0.15 | 0.02 |
| Skeletal muscle density | -0.045 | 0.48 | -0.046 | 0.47 | 0.40 | **<0.001** |
| Mediastinal adipose tissue | -0.057 | 0.37 | 0.16 | 0.01 | 0.010 | 0.88 |
| Visceral adipose tissue | 0.017 | 0.78 | 0.053 | 0.41 | 0.13 | 0.04 |
| Femoral neck density | 0.005 | 0.94 | 0.008 | 0.90 | -0.027 | 0.67 |
| Psoas volume | 0.038 | 0.55 | 0.13 | 0.04 | 0.06 | 0.34 |
| Psoas density | 0.049 | 0.44 | -0.075 | 0.24 | 0.43 | **<0.001** |
| Lower leg muscle volume | 0.10 | 0.12 | 0.099 | 0.13 | 0.16 | 0.03 |
| Lower leg muscle density | -0.10 | 0.12 | -0.028 | 0.67 | 0.18 | 0.005 |
| Upper leg muscle volume | 0.045 | 0.48 | 0.11 | 0.08 | 0.11 | 0.10 |
| Upper leg muscle density | -0.098 | 0.12 | -0.049 | 0.44 | 0.083 | 0.19 |
| **Organ Measurements** |  |  |  |  |  |  |
| Gall bladder volume | 0.014 | 0.83 | 0.066 | 0.30 | -0.14 | 0.03 |
| Heart volume | 0.11 | 0.07 | 0.091 | 0.15 | 0.19 | 0.003 |
| Kidney volume | 0.049 | 0.44 | 0.028 | 0.66 | 0.21 | **<0.001** |
| Lung volume | -0.019 | 0.76 | 0.082 | 0.19 | -0.054 | 0.39 |
| Lung density | 0.079 | 0.22 | -0.011 | 0.86 | 0.27 | **<0.001** |
| Liver volume | 0.12 | 0.05 | -0.023 | 0.72 | 0.075 | 0.24 |
| Liver density | 0.00 | 1.00 | -0.11 | 0.09 | -0.10 | 0.12 |
| Spleen volume | 0.096 | 0.13 | 0.025 | 0.70 | -0.044 | 0.49 |

KIDA data are given for the index knee. The p-value for 30 comparisons is adjusted to a p-value less than 0.00167.

**Supplementary Table S3**– Bone density around the index knee joint and body composition

|  | **beta** | **P-value** |
| --- | --- | --- |
| Skeletal muscle density | 0.28 | <0.001 |
| Psoas density | 0.20 | <0.001 |
| Kidney volume | -0.002 | 0.59 |
| Lung density | 0.017 | <0.001 |

Data are from multiple linear regression for the index knee bone density as measured by KIDA. Adjustment are for age, sex, BMI

**Supplementary Table S4–** WOMAC subcomponents in relation to lung, psoas and leg muscle findings

|  | **Lung volume** | | **Lung density** | | **Psoas density** | | **Lower leg muscle density** | |
| --- | --- | --- | --- | --- | --- | --- | --- | --- |
|  | **Beta** | **p-value** | **Beta** | **p-value** | **Beta** | **p-value** | **Beta** | **p-value** |
| Total WOMAC | 0.002 | 0.04 | -0.043 | 0.006 | -0.31 | 0.03 | -0.46 | 0.01 |
| WOMAC pain | 0.001 | 0.25 | -0.027 | 0.11 | -0.35 | 0.03 | -0.47 | 0.01 |
| WOMAC stiffness | 0.000 | 0.75 | -0.027 | 0.15 | -0.11 | 0.55 | -0.016 | 0.94 |
| WOMAC physical function | 0.002 | 0.02 | -0.049 | 0.002 | -0.33 | 0.03 | -0.50 | 0.007 |

Data are from multiple linear regression and adjusted for age, sex, BMI, subcutaneous fat.
